# Simultaneous multislice (SMS) imaging techniques

**DOI:** 10.1002/mrm.25897

**Published:** 2015-08-26

**Authors:** Markus Barth, Felix Breuer, Peter J. Koopmans, David G. Norris, Benedikt A. Poser

**Affiliations:** ^1^ Centre for Advanced ImagingThe University of QueenslandBrisbaneAustralia; ^2^ Radboud University Nijmegen, Donders Institute for Brain, Cognition and Behaviour, Donders Centre for Cognitive NeuroimagingNijmegenThe Netherlands; ^3^ Research Center Magnetic Resonance Bavaria (MRB)WürzburgGermany; ^4^ FMRIB CentreUniversity of OxfordOxfordUnited Kingdom; ^5^ Erwin L. Hahn Institute for Magnetic Resonance Imaging, UNESCO‐Weltkulturerbe ZollvereinLeitstand Kokerei ZollvereinEssenGermany; ^6^ MIRA Institute for Biomedical Technology and Technical MedicineUniversity of TwenteEnschedeThe Netherlands; ^7^ Department of Cognitive Neuroscience, Faculty of Psychology and NeuroscienceMaastricht UniversityMaastrichtThe Netherlands; ^8^ Maastricht Brain Imaging Center (M‐BIC)Maastricht UniversityMaastrichtThe Netherlands

**Keywords:** simultaneous multislice imaging, multiband imaging, fast imaging

## Abstract

Simultaneous multislice imaging (SMS) using parallel image reconstruction has rapidly advanced to become a major imaging technique. The primary benefit is an acceleration in data acquisition that is equal to the number of simultaneously excited slices. Unlike in‐plane parallel imaging this can have only a marginal intrinsic signal‐to‐noise ratio penalty, and the full acceleration is attainable at fixed echo time, as is required for many echo planar imaging applications. Furthermore, for some implementations SMS techniques can reduce radiofrequency (RF) power deposition. In this review the current state of the art of SMS imaging is presented. In the Introduction, a historical overview is given of the history of SMS excitation in MRI. The following section on RF pulses gives both the theoretical background and practical application. The section on encoding and reconstruction shows how the collapsed multislice images can be disentangled by means of the transmitter pulse phase, gradient pulses, and most importantly using multichannel receiver coils. The relationship between classic parallel imaging techniques and SMS reconstruction methods is explored. The subsequent section describes the practical implementation, including the acquisition of reference data, and slice cross‐talk. Published applications of SMS imaging are then reviewed, and the article concludes with an outlook and perspective of SMS imaging. Magn Reson Med 75:63–81, 2016. © 2015 The Authors. Magnetic Resonance in Medicine Published by Wiley Periodicals, Inc. on behalf of International Society of Medicine in Resonance.

## INTRODUCTION

The possible advantages of simultaneously exciting and imaging several slices were realized early in the development of MRI 1. Already in 1980, simultaneous multislice (SMS) excitation was proposed as a method of improving the efficiency of line‐scan imaging techniques 1. Improved understanding of the physics of slice selective excitation led Müller 2 to propose the application of the Fourier shift theorem to generate the radiofrequency (RF) pulses necessary for SMS excitation. At a time in which single channel RF coils were almost universally used for MR signal reception the main possibility open to these early workers for disentangling the superposed signals from the acquired lines 1 or slices 2 was the phase manipulation of the signal. Initially schemes such as Hadamard encoding were used 1, 3, but to disentangle the signals from *N* slices, the imaging experiment must be repeated *N* times, with a different phase pattern for each excited slice. The use of such add/subtract schemes makes the experiment vulnerable to the effects of motion and other system instabilities; furthermore, the acquisition durations become quite long. The use of Hadamard encoding was thus more attractive for spectroscopy than imaging, as the extra acquisitions necessitated generally would not exceed the number of averages required to achieve sufficient sensitivity 4, 5. Apart from using the phase some early work also showed that it is possible to use frequency‐encoding to disentangle the slices 6. This, however, has two obvious disadvantages: the frequency‐encoding direction is then oblique to the slice axis so that the voxels have a parallelogram cross‐section, and the number of data points along the frequency‐encoding direction must also be increased to accommodate the data from all the slices, thus increasing the readout duration.

An important development, that indeed foreshadowed many of those made in recent years, was the introduction of the POMP technique [phase offset multiplanar volume imaging 7]. Here multiple slices were excited, but rather than manipulating the phase of the entire slice, as in the Hadamard based techniques, each acquired slice had a unique phase gradient in k‐space that was imposed by the phase of the applied RF pulses. For example, in a two slice experiment one slice could have no phase variation, and the phase of the other would alternate between 0 and π for successive k‐space lines. This latter slice would, hence, be shifted by half a field of view (FOV) in image space, so that if the FOV in the phase‐encoding direction were doubled then the two slices would not be superimposed and could be viewed separately.

In both Hadamard and POMP imaging it was clear that the simultaneous detection of signals from *N* slices resulted in an increase in sensitivity of *√N* relative to the sequential acquisition of data from the same number of slices, but in no net reduction in acquisition time. Furthermore, it was inherent to the summation principle of the RF pulses used, that the power deposition would increase linearly with the number of excited slices, and if the pulses had identical phase then the peak power would increase with *N^2^*. The peak power could, however, be reduced by finding an optimum phase variation between the pulses 8.

**Figure 1:**
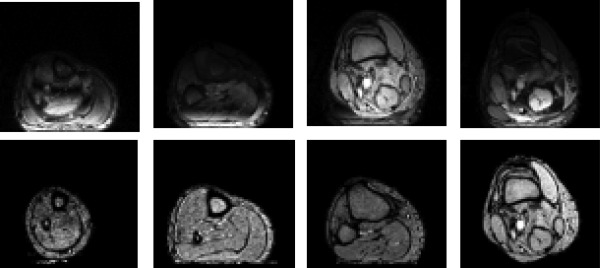
First ever in vivo SMS images obtained from the leg of a healthy volunteer using a four element coil array. The top row shows the images obtained from each coil, the bottom row shows the disentangled slices using a SENSE reconstruction. Figure taken with kind permission from Larkman et al 12.

The revolution in imaging acquisition brought about by the introduction of parallel imaging techniques 9, 10, 11 at the turn of the millennium had an immediate impact on simultaneous multislice imaging. The possibility of disentangling simultaneously excited slices by means of the coil sensitivity profiles and, thereby dramatically reducing acquisition times, was initially demonstrated by Larkman et al in 2001 12 (Fig. 1). However, this idea was only followed up by a relatively small community, and did not attract widespread attention for almost a decade. Contributing factors to this slow development were probably the relatively late availability of receiver coils with a coil distribution along the *z*‐axis, which is necessary to accelerate axial acquisitions; and the lack of an obvious application. Despite this relative lack of initial interest, important progress was made during these years, and special attention should be given to the CAIPIRINHA (controlled aliasing in parallel imaging results in higher acceleration) technique 13, which in a similar manner to POMP uses the phase of the RF pulses to shift the position of adjacent slices in image space. This makes it far easier to disentangle adjacent slices where the coil sensitivity profiles are similar; hence, the gap between slices can be reduced.

For most pulse sequences, the accelerations achievable by implementing standard parallel imaging and SMS imaging are similar, but the implementation of SMS is more demanding as higher power RF pulses have to be incorporated into the imaging sequence. A significant advantage of SMS is that it does not directly cause a reduction in sensitivity, whereas this is inherent to the reduction in the number of phase‐encoding steps acquired in standard parallel imaging methods. For echo planar imaging (EPI) in particular, the benefits of SMS are qualitatively different than for standard parallel imaging. The latter reduces the duration of the EPI readout; hence, both the distortion and the SNR are correspondingly reduced. SMS leads to a reduction in acquisition time by a factor N, with no impact on distortion or SNR. Although an earlier abstract had demonstrated the combination of EPI with SMS 14, it was the subsequent publications of highly accelerated EPI images by Moeller et al 15, (initially in abstract form 16, who also introduced the term “multiband”) and Feinberg et al 17 that drew widespread attention to this technique. The development of more powerful and robust reconstruction techniques 15, 18, combined with the invention of blipped CAIPIRINHA 18, that brought the benefits of CAIPIRINHA to EPI, set the stage for the widespread implementation and use of SMS techniques, including the human connectome project 19. We shall use the term “SMS” to refer to the technique in general, and the multiband factor “MB” as a descriptor for the number of simultaneously acquired slices, or the RF pulses used for simultaneous excitation.

In this review, we shall give a comprehensive and detailed explanation of current techniques for performing SMS imaging, in so doing we build upon a previous related review 20, and also a virtual issue of this journal on Simultaneous Multislice Imaging. We shall also chart the continuing expansion of this methodology to an ever widening field of applications.

## SMS RF PULSES

A slice selective complex RF pulse can be described as the product of two functions:

(1) RF(t)=A(t)⋅P(t)

where *A(t)* is the standard complex RF waveform that in conjunction with the slice selective gradient determines the slice *profile* (e.g., a sinc or hyperbolic secant), and *P(t)* is an additional phase modulation function that determines the slice *position* (Δω) and its phase (φ) at TE = 0 according to:

(2) $$P\left(t\right)=e^{i\Delta \omega t+\varphi \;}.$$

The simplest way to obtain SMS excitation is to sum multiple RF waveforms with different *P(t)* resulting in a multiband pulse that excites the desired slices in the presence of a common slice selective gradient.

(3) $$RF_{MB}\left(t\right)=A(t)\cdot \underset{N}{\sum }e^{i\Delta \omega_{n}t+\varphi_{n}}.$$

Note that *A(t)* remains unchanged in most common situations where the same flip angle and slice profile are desired for each slice, which will be assumed for the remainder of this section. However, this is not a general requirement, and in principle *N* arbitrary waveforms at arbitrary slice positions can be added up by complex summation to form a multiband pulse, as long as each pulse individually is consistent with the chosen slice selection gradient. Slice and transmit channel dependent pulse shapes *A_n_(t)* play an important role in parallel transmission (pTX) SMS excitation which will be discussed below.

There are two major technical challenges with MB RF pulse design. *RF_MB_(t)* is prone to exceed peak amplitude capabilities of the RF amplifier and the total power in the pulse may exceed specific absorption rate (SAR) limits. Increasing the duration of the RF pulse—while keeping the flip angle and bandwidth‐time‐product (BWTP) constant to ensure identical slice profiles—will reduce both SAR and peak amplitude. Unfortunately, the amount of stretching needed for high MB factors can have serious implications with respect to sequence timing, robustness against off‐resonance effects (due to reduced bandwidth) and non‐negligible T_2_^*^ decay during the pulse. Alternative strategies are discussed below, starting with methods to reduce peak amplitude without affecting SAR, followed by methods that achieve time‐averaged power reduction.

### Peak Amplitude Scaling and Reduction Methods

Strictly speaking, the peak amplitude of *RF_MB_(t)* is governed by the amplitudes of both *A(t)* and *ΣP_n_(t)*. In practice, however, *ΣP_n_(t)* will be oscillating at such a high frequency compared with the modulations in *A(t)* that one can approximate max(|*RF_MB_(t)*|) to be equal to max(|*ΣP_n_(t)*|) × max(|*A(t)*|).

If *φ* is identical for all slices, max(|*ΣP_n_(t)*|) simply scales with *N*. Lower peak amplitudes can be achieved by allowing the phase of each slice to vary 21. Numerical simulations have shown that, at large *N*, max(|*ΣP_n_(t)*|) approaches its theoretical lower bound of sqrt(*N*) and that for acceleration factors in the range of four to eight the theoretical optimum is exceeded by 24 or 14%, respectively 22, 23. When implementing an RF phase‐cycling scheme for CAIPIRINHA in segmented acquisitions (see the section on SMS sampling strategies), care must be taken that none of the steps in the scheme exceeds the maximum RF amplifier output.

An alternative strategy to reduce peak amplitude in spin‐echo imaging was recently proposed by Auerbach et al 24 similar to earlier work by Goelman 25. Multiple slice‐selective pulses were shifted in time by 1–2 ms such that their peaks no longer overlapped. Whereas this can increase the total duration of the pulse quite dramatically for high accelerations factors, these pulses do not suffer from reduced bandwidths, as effectively each slice is simply excited separately in time. Time‐shifted RF pulses in the context of SMS imaging had been demonstrated previously 26, 27 in a technique sometimes referred to as “multiplexing,” but in those studies the goal was to temporally shift the signals to be able to disentangle them as opposed to reducing peak RF amplitude. The major downside of the time‐shifted strategy is that the slice selection gradient that is needed for the later slices dephases the earlier ones. As such, there is no refocusing gradient that can rephase all slices. This limits the method to applications that use more than one RF pulse, e.g., a spin‐echo sequence. In which case there are two options: (a) The echoes of all slices coincide meaning the echo times vary (by twice the amount of the RF pulse time shifts), and (b) All echo times are identical but the occurrence of the echoes is shifted by the same amount as the RF pulses. Note that the latter option also requires that the excitation bandwidth is double that of the refocusing pulse, increasing the power requirements of the excitation pulse.

A method closely related to the time‐shifting strategy is that of “root‐flipping” for MB RF pulses using a SLR design approach 28. Effectively, time‐shifting is achieved without lengthening of the total pulse duration by creating asymmetric RF‐pulses such that, when added, their peaks do not overlap. The same problems with varying echo times occur, however, and because asymmetric RF pulses do not have a linear through‐slice phase ramp but a quadratic phase profile, here too the requirement is that the composite pulses are applied in pairs.

### Power Scaling and Reduction Methods

The methods described above aim to reduce the peak amplitude so as to allow the RF pulses to be produced by the amplifiers without clipping. However, with high multiband factors and/or high flip angles, power deposition in the body becomes the main limitation. Irrespective of the manipulations outlined above, the power of multiband pulses scales with *N*. This can be shown using Parseval's theorem:

(4) $$\overset{\infty }{\underset{-\infty }{\int }}{\left|\theta \left(k\right)\right|}^{2}dk=\overset{\infty }{\underset{-\infty }{\int }}{\left|\Theta \left(z\right)\right|}^{2}dz\;.$$

In terms of multiband design, Eq. (4) means that the total power in image/frequency space (power integral of the [multi‐] slice profile) is proportional to the total power in the excitation k‐space description 29. In other words: regardless of the exact phase‐modulated shape of *RF_MB_(t)*, if the number of slices in image space is altered (i.e., changing *N* in Eq. (3)), the RF power increases *by the same factor*. Please note that this is only valid for sets of *P_n_(t)* that do not create overlapping slices, but this requirement is generally met in SMS applications.

Parseval's theorem then appears to put quite a hard limitation on power reduction, because there is not much leeway in how we want to distribute our slices. The crucial degree of freedom, however, is given by *how* we traverse excitation k‐space, or—put another way—how the k‐space trajectory is mapped onto the time domain.

### Variable Rate Excitation (VERSE)

The true RF power is determined by the power integral of the RF over time. By varying the slice selection gradient with time, the k‐space representation of the RF pulse is no longer mapped linearly to the time domain, and power can be reduced without modifying the slice profile. This is the principle of VERSE 30. Power reduction is achieved by slowing down k‐space traversal at coordinates where most energy needs to be deposited (i.e., the peak of the [multiband] RF waveform) by temporarily reducing the amplitude of the slice selection gradient. Time lost by doing this can be recovered by speeding up at times of low RF amplitude. VERSE has great potential to reduce the power of a pulse but is very sensitive to off‐resonance effects 30. Whereas a standard RF pulse off‐resonance only experiences a slice shift, the sensitivity of a VERSE pulse to off‐resonance varies with time due to the varying ratio between the gradient strength and the magnitude of the local field inhomogeneity, which can lead to a corrupted slice profile as demonstrated in the original study 30. In practice, the low extent to which VERSE needs to be applied to 180^°^ refocusing pulses with moderate multiband factors (MB < 4) at 3 Tesla (T), still allows an acceptable slice profile and high effective bandwidth‐time product 31.

### Power Independent of the Number of Slices (PINS)

**Figure 2:**
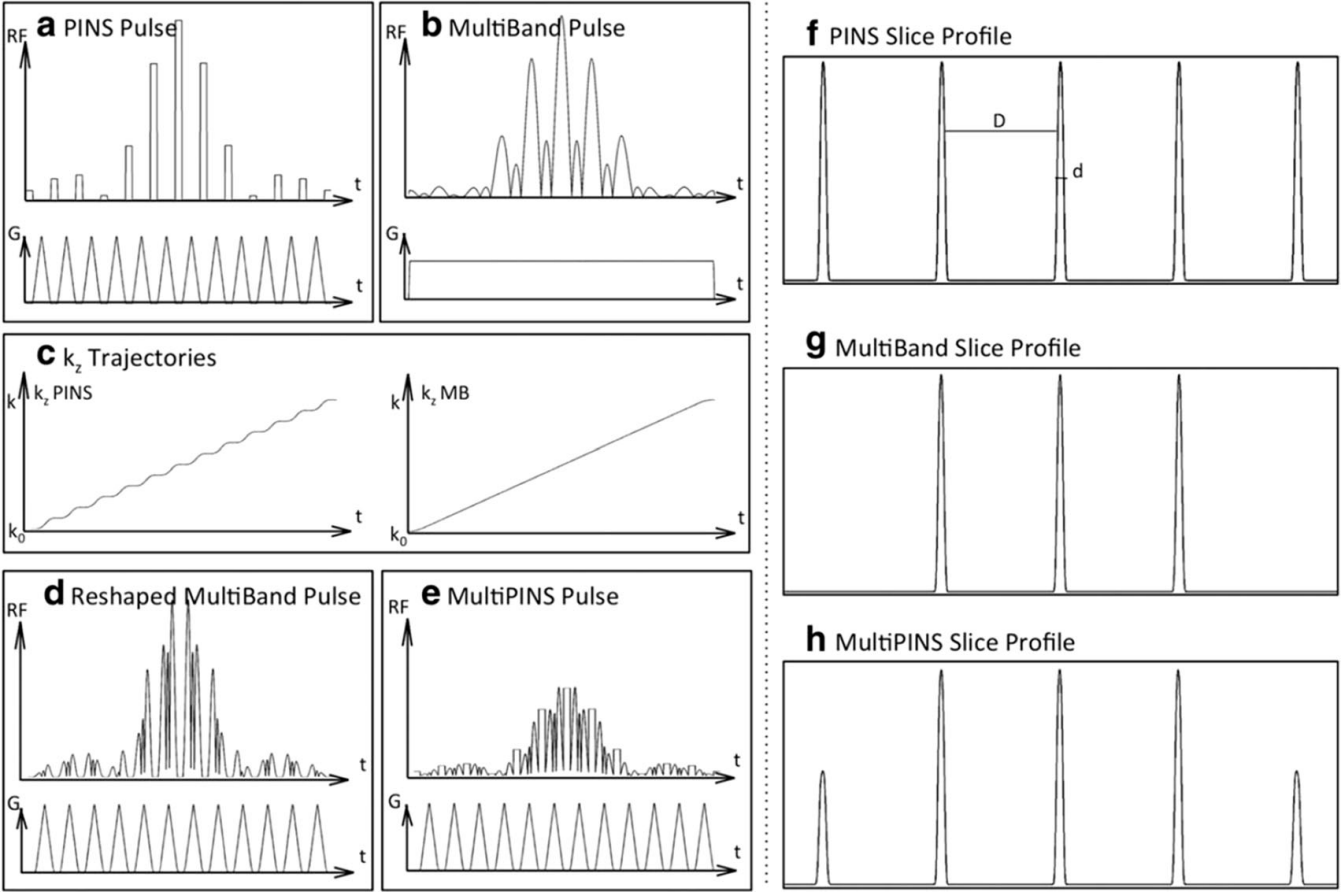
PINS, multiband and multi‐PINS RF pulses. The RF envelope, its accompanying gradient and the slice profile for a PINS pulse (**a,f**) and a conventional multiband pulse (**b,g**). Corresponding k‐space trajectories are shown in (**c**). Multi‐PINS works by summing a PINS and a multiband pulse, but as they have different gradients VERSE needs to be applied to the multiband pulse to allow it to operate with the blipped gradients (**d**). Finally, adding them results in the multi‐PINS pulse in (**e**) with (**h**) the resulting slice profile. Figure taken with kind permission from Eichner et al 40.

Norris et al proposed the use of periodic excitation profiles for SMS imaging to reduce RF power in a method called PINS 32, 33. Series of nonselective rectangular RF pulses are interleaved with small slice gradient blips (see Figure 2a). In k‐space, this equates to depositing a specific amount of RF energy at a single coordinate, stopping transmission and moving to the next desired location in k‐space before starting transmission again. The power deposition at a set of discrete k‐space coordinates can be seen as sampling the original RF waveform by multiplying it with a train of delta functions. After a Fourier transform, this equates to convolving the single‐slice profile with another train of delta functions, creating a periodic series of slices, where the periodicity is determined by the area of the gradient blips. With respect to Parseval's theorem, PINS can be seen as a special case of VERSE in which an infinite power in k‐space (corresponding to an infinite number of slices) is mapped to a finite power in the time domain by the stratagem of turning off the gradients during the application of the RF pulse. An interesting consequence of this is that PINS pulses have an exceedingly low magnetisation transfer contrast effect, as the RF pulses are always transmitted on‐resonance 34. Periodic excitation has been used before to create cardiac tagging patterns 35, 36 and dates as far back as the mid 1970s 37 when trains of rectangular RF pulses of variable durations separated by periods of free precession were used to perform frequency selective excitation in NMR, as Fourier transform‐based waveforms were hard to compute and used up valuable data storage space. In this case, the additionally excited frequency bands were considered an unwanted by‐product). Later, such pulses were developed in binomial expansion for solvent suppression 38.

The bandwidth of a PINS pulse is of limited magnitude: the bandwidth of an RF pulse is proportional to the average slice selection gradient amplitude. With PINS, the gradient blips are typically small while being interleaved with periods of no gradient at all when an RF sub‐pulse is transmitted. The BW of a PINS pulse can be readily calculated from Eq. (4) in Koopmans et al 32 as:

(5) $$BW=\frac{Slice\;Thickness}{Slice\;Period*(BlipDuration+SubpulseDuration)}.$$

This means that particularly in high‐power applications with small slice spacing the bandwidth can be fairly low, which can lead to slice distortions in the presence of B_0_ inhomogeneities. Although this is not ideal, it should be put in perspective: virtually all EPI studies to date have suffered from distortion in the phase‐encoding direction to a much larger extent without it being prohibitively bad.

This leaves the question when to use PINS and when to use a conventional, higher BWTP (VERSE) multiband pulse. This depends on the application, with PINS being most useful if the multiband factor is high and/or slices are relatively thick with respect to the slice period. PINS is least useful in the opposite case when the slices are thin relative to their spacing as Eq. (5) dictates that to obtain an acceptable BWTP one would need a short interval between the sub‐pulses, which will generally be constrained by the gradient slew rate. To illustrate: Eichner et al 39 investigated the relationship between SAR and multiband factor for PINS and both VERSE and normal multiband pulses for slices of 1.5 mm thick and a matched BWTP of 2. For an MB factor of 2, the SAR was very similar for all pulses, for an MB factor of 3, multiband pulses used only 25% more power than PINS, but for an MB factor of 5 this value was over 200%. As a rule of thumb, a PINS gradient blip takes approximately 70–120 μs depending on the slice spacing and slew rate. In high resolution studies, the thickness/periodicity ratio tends to be around 1/30, meaning that 60 sub‐pulses are required to achieve a BWTP of 2, i.e., approximately 6 ms are spent on gradients. Many SMS spin‐echo brain studies use 10 ms for the refocusing pulses 19, 24, 34 and in practice, the remaining 4 ms of hard‐pulse transmission in PINS is sufficient to achieve 180 flip angles without running into SAR problems.

PINS produces periodic slice profiles meaning that in theory the slice FOV is infinitely large. This can create difficulties in cleanly reconstructing all the slices; however, there are several strategies available for avoiding these. As PINS is primarily used in high‐power applications like spin‐echo, one can simply use conventional SMS excitation pulses to limit the FOV and only apply PINS to the refocusing pulses that need power reduction most. If PINS is needed for excitation as well, a sagittal orientation can be a solution when imaging the brain, as the additional slices then fall outside the head. Finally, one can opt to exploit limited coil coverage by trying to position the unwanted slices outside the range of either the transmit or receive arrays.

### Verse Multiband Pulses Combined with Pins: Multi‐Pins

To improve the BWTP and power performance of PINS for refocusing pulses in a spin‐echo sequence, Eichner et al have proposed a hybrid technique dubbed multi‐PINS 40, shown in Figure 2e. Here, a PINS pulse (Fig. 2a) and a conventional multiband pulse (Fig. 2b) are added together, the conventional multiband pulse being transmitted during the presence of the blips (while applying VERSE to the multiband pulse to correct for the time‐varying amplitude of the gradient blips as shown in Figures 2c,d). This increases the power efficiency of the overall pulse as now RF is also transmitted during the periods of gradient blips. This in turn allows one to reduce the duration of the RF subpulses which, as seen in Eq. (5), leads to a higher bandwidth.

The slices affected by the multiband pulse will receive the summed flip angle of both techniques, whereas the remaining periodic slices will only receive the flip angle imposed by the PINS pulse. Like PINS, multi‐PINS is mostly used in a spin‐echo sequence for the refocusing pulse, while excitation is performed with a conventional multiband pulse (see Figures 2f–h) so that the outer slices are not imaged.

### Parallel Transmission and SMS

Parallel RF transmission plays an increasingly important role at ultrahigh field and presents a way of dealing with inhomogeneity in the B1+ RF transmit field 41, 42. A practical, and often more power efficient way forward than single‐channel transmission is the use of local transmitter arrays where each transmit channel is driven independently, allowing channel dependent control of RF amplitude and phase, or pulse waveform 42, 43. The transmission can then for example be tailored to homogenize B1+ in a slice by slice manner 44, while reducing overall power deposition.

The advantages of pTX directly extend to multislice excitation. The considerations for SMS do not in principle differ from those for single slice pulses, because multiband pulses can simply be generated by complex summation of individually designed single‐slice waveforms over the same gradients, as explained above. Early work on SMS‐pTX made use of this to achieve multislice excitation with a slice‐specific B1+ shim 45, 46, and it has been shown that considerable SAR reductions can be achieved when using suitable transmit coil geometries such as multi‐row (“z‐stacked”) designs 45, 47, 48, 49. Multiband pTX pulses with slice‐specific shims have also been applied to diffusion weighted spin‐echo EPI acquisitions at 7T 50. Especially at high field and for large flip angle pulses, management of peak power on all channels, and SAR is crucial. For multiband pTX pulses, this can be achieved in ways analogous to regular multiband pulses, most notably by varying the phase of the individual slice excitations. This principle has been used in recently developed “integrated” multiband pTX single and multispoke pulse designs that simultaneously optimize for peak power and local SAR 48, 51.

As an alternative to B1+ shimmed multiband pTX pulses, Sharma et al. have proposed to merge the PINS 33 and kt‐points 52 techniques by augmenting the PINS trajectory with x‐y blipped sub‐pulses away from the origin to achieve a more homogeneous SMS excitation 53. The spatial resolution of the B1+ shim in what is effectively a volume excitation, however, is rather limited, and a trade‐off has to be made between the B1+ homogenization and very long B0 sensitive pulses. The additional x‐y blipped PINS subpulses for B1 shimming also reduce the number of primary slice‐encoding subpulses possible within an acceptable total pulse duration. The use of off‐center subpulses, furthermore, results in a variable and a reduced time‐averaged slice‐selection gradient, which gives an even lower pulse bandwidth combined with a VERSE‐like off‐resonance response.

### SMS and Cross‐Talk/Partial Saturation

**Figure 3:**
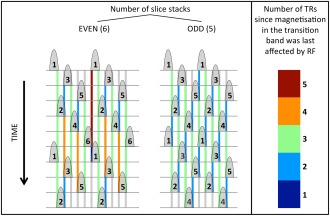
Cross‐talk saturation in SMS imaging. The schematic shows a series of interleaved nonideal multiband slice profiles that overlap slightly due to BWTP limitations. The numbers inside the slices indicate the index of each slice group, the top row shows the first excitation (t = 0) with time being displayed vertically downward. For a slice that is about to be excited, the colored rectangles indicate when its transition bands have last been partially saturated by the neighboring slice profiles. The colorbar on the right indicates the number of TRs that each color represents. The left part of the figure shows the schematic corresponding to an even number of interleaved slice groups, the right shows the same for odd‐numbered excitation schemes. The scheme on the left is highly inhomogeneous with especially severe saturation effects in groups 1 and 6. These effects are nonexistent in the scheme on the right.

In 2D imaging, to prevent saturation effects from neighboring slices with nonideal slice profiles, an interleaved slice excitation scheme is often used to allow for as much relaxation as possible (half a volume TR). In SMS imaging, this is still possible albeit with a small caveat: the number of stacks (i.e., total number of slices divided by the MB factor) needs to be an odd number 31. If it is even the last excitation of the first TR will affect the magnetization of the first excited slice in the second TR; hence, the saturation artifacts would be maximized. If an odd number of stacks is used, this does not occur (see Figure 3).

## SMS SAMPLING AND RECONSTRUCTION STRATEGIES

There are three ways in which SMS spatial encoding can be accomplished: RF phase encoding, gradient phase encoding and coil encoding. In practice, modern SMS acquisitions will use a combination of coil encoding with either RF encoding or gradient encoding to resolve the slice direction (i.e., RF and gradient slice encoding are in many cases interchangeable). The in‐plane direction is sampled with readout and phase encode gradients as in a regular 2D slice‐selective acquisition. Furthermore, it is generally the objective to acquire the simultaneously excited slices in such a way that they appear shifted with respect to each other, which aids the subsequent reconstruction as discussed later in this section. For clarity, we first introduce the various relative slice shift approaches and then demonstrate how they can complement the slice‐encoding method for efficient SMS acquisitions.

### (1). RF Phase Encoding

**Figure 4:**
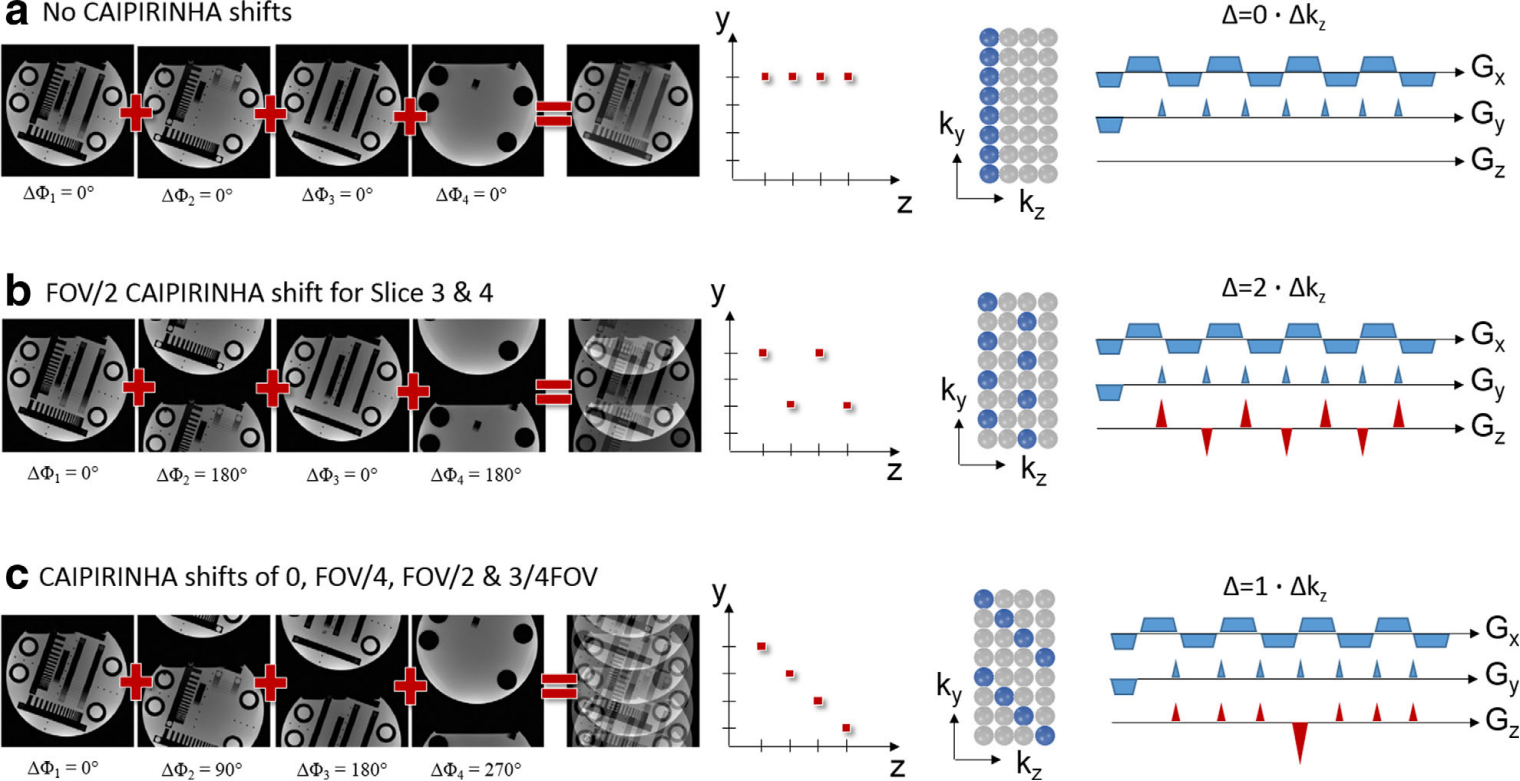
Analogy of phase cycled CAIPIRINHA and blipped CAIPIRINHA: 4 Slices are simultaneously excited. **a:** No phase cycling causes the slices to overlap directly on top of each other. This corresponds to a standard SMS EPI acquisition without using gradient blips along the z direction. **b:** 180° phase cycling used for slice 2 and 4. Slice 2 and 4 appear shifted by FOV/2 in the FOV. The same aliasing pattern can be realized by using alternating gradient blips along the z direction. The gradient moment of the blips must be chosen such that Δ = 2Δk_z_. **c:** All four slices are shifted by different amounts (0,FOV/4,FOV/2,3/4FOV). By alternating between four different multiband pulses for subsequent phase encoding steps different phase cycles may be imposed for each individual slice. Alternatively, gradient blips may be used on the z‐axes such that subsequent phase encoding steps accumulate a phase according to 1Δk_z_. To avoid spin dephasing over the slices the accumulated gradient moments are rephased accordingly. All the sampling schemes can be represented in k‐space with sampling positions in k_y_ ‐ k_z_ space in analogy to 2D CAIPIRINHA for 3D imaging.

According to the Fourier shift theorem, an image slice can be shifted to different locations in the FOV along the phase encoding direction by imposing a corresponding phase gradient on the acquisition k‐space. One can achieve this by applying a phase gradient to the RF pulse. For multiband excitations, shifts between simultaneously excited slices can be accomplished by applying individual phase cycling patterns to each frequency band. The number of different multiband RF pulses that are required will depend on the number of simultaneous slices and the desired shifts between them as demonstrated in Figure 4. RF phase encoding was first proposed in the context of the POMP technique 7, where all *N* slices were accommodated into one large FOV without overlap (i.e., full slice encoding). This required the FOV to be a factor *N* larger than the single slice FOV and each slice to be shifted by a unique integer multiple of the FOV. This was achieved with a factor of *N* more phase encoding steps at *N* times tighter sampling distance of 1/N⋅Δk, while using alternating RF‐pulses with phases $\phi_{nk}=2\pi \cdot (k-1)\cdot (n-1)\;/(2N)$ with *n* and *k* running from 1 to N. RF encoding is directly applicable to standard “one‐line‐per‐excitation” sequences such as GRE or SE.

### (2). Gradient Phase Encoding

Simultaneously excited slices can be resolved by conventional gradient phase encoding along the slice direction. This is in direct analogy to three‐dimensional (3D) imaging where a thick slab is excited and phase encoded along the slice direction. This perspective can be taken by recognizing that a MB pulse also excites magnetization in 3D space, which naturally gives rise to the notion of a 3D k‐space 14, 54, 55. The “slice FOV” $FOV_{z}$ is then given by the number of simultaneously excited slices times the distance between them, represented in k‐space by a $k_{z}$ phase encode increment $k_{z}=1/FOV_{z}=1/(N\cdot gap)$, and the number of “voxels”; i.e., $k_{z}$‐planes is equal to N. The 3D perspective of slice encoding is equivalent to a 2D perspective with shifted slices 7, 18, and RF phase encoding is equivalent to $k_{z}$ ‐phase encoding across the slices.

### (3). Coil Encoding (Accelerated SMS Acquisitions)

Coil encoding is also known as parallel MRI (pMRI) and is an integral part of modern clinical MRI. Coil encoding almost always accompanies gradient encoding, and by replacing some of the (time consuming) conventional gradient encoding allows a substantial acceleration. In the same way, coil encoding can replace some or all of the RF or gradient slice encoding in SMS acquisitions. Larkman et al 12 were the first to perform SMS acquisitions with pure coil encoding using SENSE reconstruction (see below, and Figure 1). This was the first demonstration of what today is usually suggested by the term SMS imaging: the simultaneous acquisition of multiple slices within the time it takes to conventionally acquire one slice. In a 3D k‐space representation, this corresponds to undersampling along the $k_{z}$ direction 56. The challenges of separating simultaneous slices by means of coil encoding alone become obvious when considering that typically (a) the distance between slices can be rather small and (b) many common receiver coil array designs provide little or no encoding power along the slice axis. Typical SMS acquisitions, therefore, use a combination of coil and either RF or gradient encoding to disambiguate the slices.

### (4). Combining $k_{z}$ Phase Encoding and Coil Encoding

The limitations of coil encoding have led to the concept of CAIPIRINHA 13, which makes it possible to shift simultaneously excited slices by different amounts within the phase encoding FOV in a “controlled” manner. In the original publication 13, these shifts were accomplished by RF‐phase encoding, analogous to POMP. The implications of the freedom to arbitrarily shift the slices are considerable: one can separate slices that are very close together, or even when the coil has no encoding power in the slice direction. The improvement in conditions for coil encoding the slices can be described by the following equivalent statements, that the shift: (a) reduces overlap between aliased slices; (b) increases distance between overlapping voxels; (c) and causes the slice separation to rely partially (or fully) on coil sensitivities along the in‐plane phase‐encoding direction.

Thus, by using suitable shift patterns, the degree of coil sensitivity encoding can be shared arbitrarily between the two directions ( $k_{y}$, $\;k_{z}$), as can be seen by reference to Figure 4. As a consequence, the notion of an “undersampling factor” along one or the other direction in the common parallel imaging sense is lost, and the acquisition is more appropriately described by the total undersampling factor and a shift pattern 57.

Soon after the original SMS‐CAIPIRINHA publication, the same principle was transferred to volumetric 3D acquisitions 57. Because only a single thick slab is excited, RF phase encoding is not an option and controlled aliasing is achieved by additional gradient moments on the z‐axis.

The ability to invest coil encoding along one direction to undersample along another has had the most impact for pulse sequences where in‐plane $k_{y}$ undersampling resulted in no or little reduction of the acquisition time. This is particularly relevant for EPI applications where a fixed TE is required: in fMRI to allow the BOLD contrast to develop, or in diffusion‐weighted EPI to include the diffusion encoding module. The temporal slice acceleration achieved is equal to the nominal slice undersampling factor, resulting in 2D EPI scans with drastically reduced acquisition times. These benefits have also been translated to RARE sequences as will be discussed later 34, 58.

SMS‐CAIPIRINHA experiments with EPI were first demonstrated by Nunes et al 14. The slice shift was produced by a train of unipolar gradient blips on the slice axis. This imposed the desired phase between k‐space lines, but the limitation was a phase accumulation along the EPI readout that effectively dephased the spins across the slice. This was solved by Setsompop et al 18, with a subtle but crucial modification that uses blipped rewinder gradients to keep the phase accumulation within the [‐π, π] range, while imparting the same phase difference between adjacent k‐space lines. This method, termed blipped‐CAIPI greatly impacted the way EPI acquisitions were carried out and immediately found widespread acceptance for BOLD‐ and diffusion‐weighted imaging.

### SMS–pMRI Reconstruction Methods

In pMRI, conventional gradient encoding is partially replaced by coil sensitivity encoding. Undersampling of k‐space corresponds to a reduced FOV and leads to fold‐over artifacts in the image space. This lack of gradient encoding can be compensated by using multiple receiver coils with sufficient spatial encoding capabilities: Image signals that are aliased into one pixel can be separated by exploiting the sensitivity information inherent to the receive coil array. The same concept can be used to separate the overlapping slices of an SMS acquisition, provided the coil array provides sufficient encoding. To introduce the basics of various SMS parallel image reconstruction strategies, we begin by reviewing conventional SENSE 10 and its adaption to SMS acquisitions as used by Larkman et al 12.

### Sense

The SENSE approach uses explicit knowledge of the spatial sensitivity information provided by each receiver coil element to perform the reconstruction in the image domain, using a pixel by pixel matrix inversion of the coil sensitivity matrix C to unfold the aliased pixels. In Cartesian, regular R fold undersampling C is a L × R rectangular matrix composed of the spatial sensitivity information from each of the L coils at the corresponding R aliased in‐plane spatial locations. The quality of the unfolding process can be described by the so‐called geometry g‐factor that quantitatively characterizes the noise amplification after SENSE reconstruction in each pixel. The more different and independent the sensitivities of aliased pixels in matrix **C** are, the lower the noise amplification will be.

In an SMS acquisition without slice shift, a scenario is created where *N* image pixels from the simultaneously excited slices are aliased directly on top of each other. This corresponds to the special case where the acceleration/reduction factor R equals the number of simultaneously excited slices *N*.

**Figure 5:**
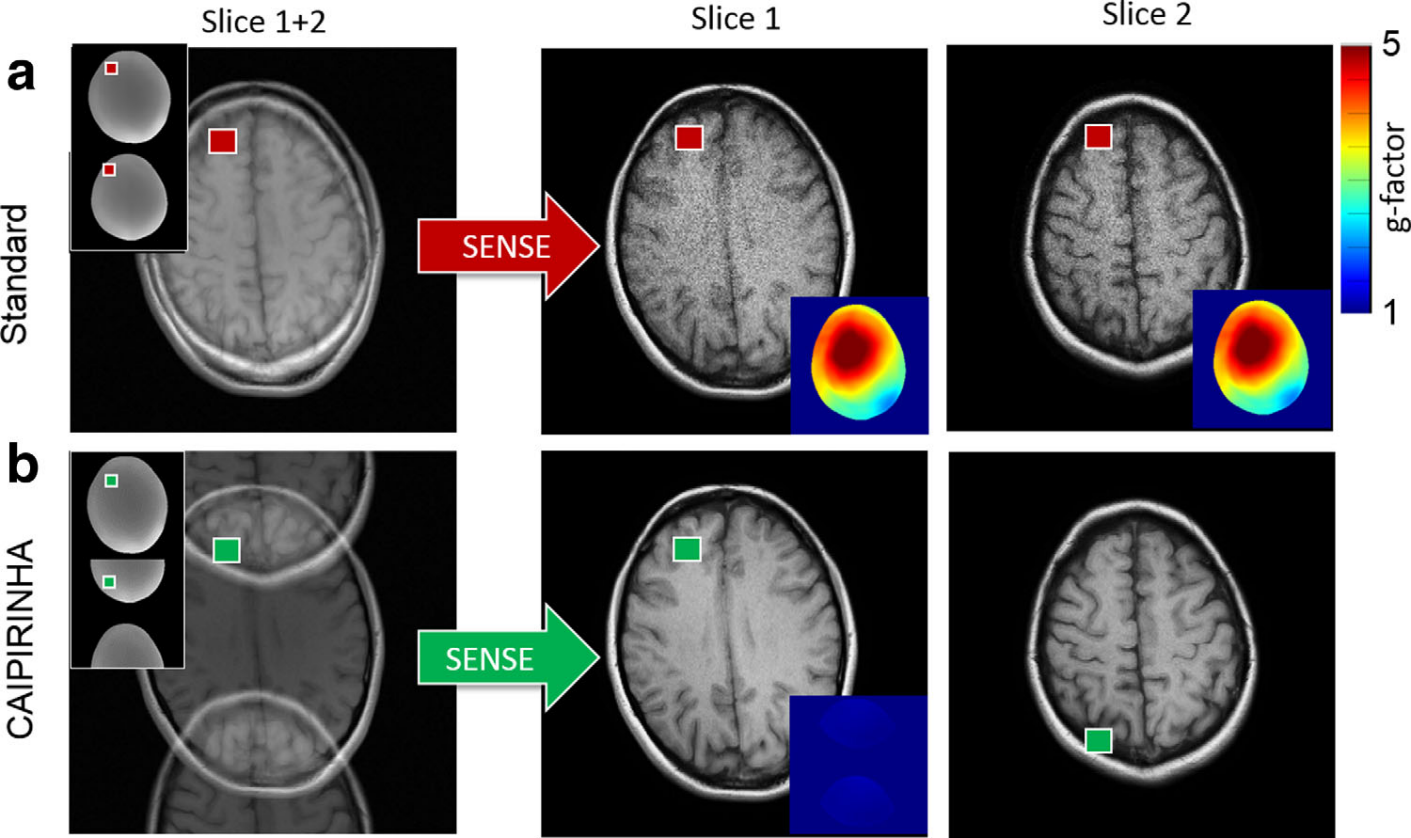
Simultaneous two slice experiment at acceleration factor R = 2 without sequence modification (**a**) and using a FOV/2 CAIPIRINHA shift for the second slice (**b**) either accomplished by RF phase encoding or gradient encoding. The folded slices in A and B can be disentangled using for example standard 1D SENSE. To this end the sensitivity maps of the individual slices are arranged along the phase encoding direction accounting for relative shifts in the case of CAIPIRINHA. As in (a), both slices are directly superimposed on top of each other the parallel imaging algorithm relies on sensitivity variations along the slice direction only. In the case of insufficient coil sensitivities along the slice direction (e.g., small slice distance) the reconstructed images suffer from large g‐factor noise enhancement. In (b), the superimposed slices appear shifted by FOV/2 with respect to each other. This allows the pMRI reconstruction (here SENSE) to use sensitivity variations along both the phase and slice directions resulting in significantly lower g‐factor noise in the reconstructed images.

Hence, the SENSE concept can be applied to SMS imaging by considering the aliasing conditions along the slice direction when populating the sensitivity matrix C. Additional in‐plane undersampling can be included into the SENSE matrix, in which case the reconstruction is performed simultaneously along the slice and phase‐encode direction in analogy to 2D SENSE 56. All parallel imaging methods are limited by the fact that aliased pixels must have sufficient sensitivity variations to perform the matrix inversion. This is crucial for undersampled SMS imaging as insufficient sensitivity variations along the slice direction can easily occur. This ill‐conditioning of the inverse problem then results in a large g‐factor noise enhancement as demonstrated in Figure 5a.

Compared with conventional in‐plane pMRI where at least at small acceleration factors (R = 2–3) aliased pixels are usually sufficiently far apart that the coil sensitivity variations make it possible to separate them, the situation in an SMS experiment can be much more challenging: consider for example an axial SMS brain scan with three simultaneously excited slices and 120 mm coverage along the slice direction: aliased pixels are then only 40 mm apart. In standard sequential MS with a phase encoding FOV of typically 220 mm and R = 3 acceleration along the PE direction, the separation of aliased pixels is much greater (∼70 mm), approximately a factor of 2 compared with the SMS acquisition. Even today, most coil arrays may not provide enough sensitivity variation along the slice direction to compensate for this, which inevitably results in high g‐factor noise in the reconstructed slices.

CAIPIRINHA 13, 57 can be used to address this limitation. The situation depicted in Figure 5a can be avoided by shifting the individual slices with respect to each other. Evidently, the aliased image pixels originate now not only from different slices but also different locations along the phase encoding direction (Fig. 5b). The coil sensitivities are typically more different in this situation and the g‐factor penalty will be reduced. As demonstrated for SMS and 3D imaging 13, 57, this can result in improved SNR and pMRI reconstructions compared with non‐CAIPIRINHA acquisitions. As a consequence, slices can now be separated even if the coil array provides no sensitivity at all along the slice direction or in applications where the slices appear very close to each other. A simple adaptation of SENSE to CAIPIRINHA sampling can be realized by adapting the sensitivity maps for the SENSE reconstruction. As sketched in Figure 5b, this can be done by virtually increasing the phase‐encoding FOV by the number of slices *N* along the phase encoding direction and shifting the individual slices such that the distance between aliased pixels is N*FOV/*R*. The procedure is shown in Figure 5 for a two‐slice experiment with *R* = 2. This “extended FOV” concept allows an arbitrary number of slices with integer undersampling factor R to be reconstructed with standard 1D SENSE. Another approach has been taken recently by using the 3D nature of the acquisition using adapted 2D SENSE 59.

### GRAPPA

GRAPPA 9 is an alternative pMRI reconstruction method which, in contrast to SENSE, uses k‐space (auto)‐calibration instead of estimating explicit coil sensitivity maps. The basic idea behind GRAPPA is to find a reconstruction weight set K, which when applied to multiple measured k‐space locations from all coils within a certain k‐space region, generates multiple nonacquired k‐space data for all the coils within this k‐space region. In the case of regular integer undersampling, these weights apply everywhere in k‐space and can thus be used to reconstruct all the missing data points in all coils. This convolution process in k‐space can also be formulated in the image space 60, 61 by a pixel‐by‐pixel multiplication of the folded multicoil images $J_{l}(x,y)$ represented in the full FOV with the GRAPPA weights $K_{kl}\left(x,y\right)\;$transformed into image space by means of inverse 2D Fourier transformation.

(6) $$I_{k}\left(x,y\right)=\overset{L}{\underset{l=1}{\sum }}K_{\text{kl}}\left(x,y\right)\cdot J_{l}\left(x,y\right).$$

The GRAPPA convolution kernel K and corresponding weights in image space K can be efficiently derived from a fully sampled low resolution reference k‐space also referred to as autocalibration signals (ACS). To this end, all the kernel replicas available within the ACS data are assembled into matrices *i* and *j* according to the missing data points and measured data points, respectively. The GRAPPA weights in k‐space are then derived by, e.g., least‐squares fitting.

(7) $$K=i\cdot {\left(j\right)}^{H}\cdot {\left(j\cdot j^{H}\right)}^{-1}.$$

In recent years, several groups have worked on implementations of robust GRAPPA based SMS reconstructions. This effort led to various different strategies for the reconstruction of SMS data with GRAPPA 18, 55, 62, 63, 64 which will be discussed in the following.

### Sense‐GRAPPA

The first GRAPPA based algorithm capable of reconstructing accelerated SMS data was the SENSE‐GRAPPA hybrid described by Blaimer et al 64. The method was originally developed for the reconstruction of regularly 2D undersampled volumetric (3D) data with GRAPPA in a one‐step implementation, as a counterpart to 2D SENSE 56. Previous GRAPPA reconstructions for 2D undersampling used a two‐step approach with successive 1D GRAPPA reconstructions along the two aliasing dimensions. The SENSE‐GRAPPA hybrid reconstruction essentially transforms the 2D reconstruction problem into a virtual 1D problem. This is highly analogous to the SMS‐SENSE approach where the sensitivity maps of multiple slices are reorganized to create a 1D problem that can be reconstructed with standard 1D SENSE. Similarly, by artificially generating reference data to obtain GRAPPA weights for an extended FOV that contains all the slices, SMS reconstruction can be performed with conventional 1D GRAPPA. This concept is straightforward to implement, and it allows both slice and any additional in‐plane undersampling to be reconstructed in a single step 63, 65.

In case of CAIPIRINHA SMS acquisitions with slice shifts, the same procedure as described above for SENSE can be applied to rearrange the reference data for SENSE‐GRAPPA reconstruction. While the slices can be separated, for certain MB factors/acceleration factors, sharp transitions of the shifted slice may appear in the full extended FOV that can cause high artifact levels in the transition region, as demonstrated in Setsompop et al 18. The reason for this is that the virtual extended ACS k‐space data for GRAPPPA calibration cannot physically be generated by any RF‐phase modulation or gradient blips scheme and thus cannot be solved in k‐space. Equivalently, the sharp discontinuities between the shifted slices cannot be adequately represented by low resolution convolution kernels in k‐space.

This limitation when simply adopting SENSE‐GRAPPA to SMS reconstruction led to the development of several alternative reconstruction approaches allowing GRAPPA to be used in combination with SMS‐CAIPIRINHA: (a) SENSE‐GRAPPA hybrid with “virtual” acceleration factors; (b) slice‐GRAPPA using slice specific reconstruction kernels; and (c) direct GRAPPA in 3D k‐space recognizing the 3D nature of the SMS acquisition.

### Sense‐GRAPPA for SMS‐Caipirinha

**Figure 6:**
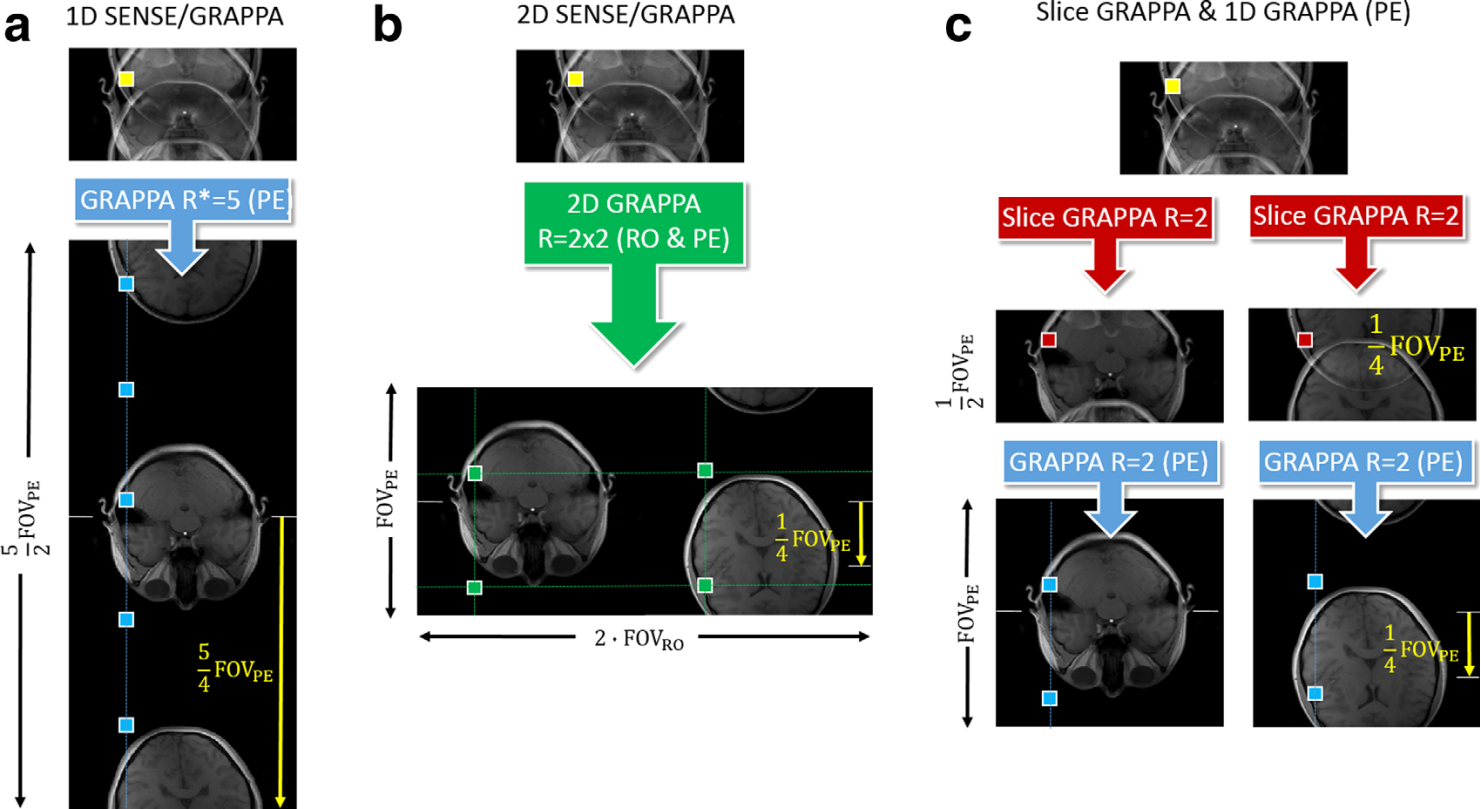
Various GRAPPA based reconstruction schemes for R = 4 SMS acquisition with acceleration along the slice and the phase encoding (PE) directions including a CAIPIRINHA shift of FOV/4 along PE. **a:** SENSE‐GRAPPA hybrid along PE: The autocalibration signals (ACS) required for the GRAPPA reconstruction are produced by rearranging the calibration data for the individual slices along an extended FOV along the phase encoding direction. The required large FOV* and the relative slice shifts have to be chosen such that R* times undersampling collapses to the actual aliasing pattern, and such that all the slices appear without overlap in the FOV*. In this case this is accomplished by choosing FOV* = 2/5*FOVPE and by shifting the second slice by 5/4FOVPE with respect to the other slice. The aliasing is finally resolved in 1 single step by using standard 1D GRAPPA along PE at R* = 5. **b:** 2D SENSE‐GRAPPA hybrid along RO & PE: Similar to A the calibration signals of the individual slices are arranged in an extended FOV, however, along the read‐out (RO) direction providing the required individual slice shifts along the PE direction. As two times in‐plane acceleration is also used, the reconstruction can be performed either in two separate 1D GRAPPA steps or in one step using a 2D GRAPPA kernel (**c**) Slice‐GRAPPA. Alternatively, the slices can be separated using slice specific GRAPPA kernels to entangle the overlapping slices. The remaining in‐plane aliasing is then resolved by 1D GRAPPA along the phase‐encoding direction in a subsequent step.

The g‐factor noise enhancement in SENSE‐GRAPPA in conjunction with CAIPIRINHA shifts described in Setsompop et al 18 can be overcome by using the following trick when generating the extended FOV ACS data for GRAPPA reconstruction 63, 65. A scenario must be created such that (a) regular integer undersampling by $R^{*}$ along the phase encoding direction in an extended FOV, here denoted by $FOV^{*}$, collapses to the actual SMS‐CAIPIRINHA aliasing pattern; (b) the individual slice shifts in the large FOV* are represented by linear phase ramps across the large FOV k‐space; and (c) all the slices appear without overlap within the extended FOV*. The procedure for meeting these conditions is described for an exemplary situation in Figure 6a.

As in the simple SENSE‐GRAPPA hybrid, the reconstructed slices appear unaliased in the extended FOV. This concept allows reconstruction of accelerated SMS acquisitions with arbitrary numbers of slices and both slice and in‐plane undersampling, by using standard 1D GRAPPA. Alternatively, as has been shown recently, the slices can also be distributed along the read‐out direction with each slice carrying the corresponding individual CAIPIRINHA shifts along the phase encoding direction 66, 67. With this approach, 2D GRAPPA can be used to directly disentangle the slices and in‐plane aliasing (Fig. 6b) or alternatively the problem can be solved in two separate 1D GRAPPA steps (not shown).

### Slice GRAPPA

Another way to reconstruct SMS‐CAIPIRINHA data with GRAPPA is the slice‐GRAPPA (SG) method 18 which uses slice‐specific GRAPPA and can thus be written as

(8) $$I_{\text{kz}}(x,y)=\overset{L}{\underset{l=1}{\sum }}K_{\text{klz}}(x,y)\cdot J_{l}(x,y)$$

with

(9) $$J_{l}\left(x,y\right)\left(x,y\right)=\overset{S}{\underset{s=1}{\sum }}J_{\text{ls}}\left(x,y\right).$$

$K_{z}$ are slice‐specific GRAPPA weights in the image domain which when applied to the aliased image to extract the signals originating only from the corresponding slice z. The k‐space weights $K_{z}$ can be calibrated from the multislice ACS from

(10) $$K_{z}=i_{z}\cdot {\left(\overset{S}{\underset{s=1}{\sum }}j_{s}\right)}^{H}\cdot {\left(\left(\overset{S}{\underset{s=1}{\sum }}j_{s}\right)\cdot {\left(\overset{S}{\underset{s=1}{\sum }}j_{s}\right)}^{H}\right)}^{-1}$$

where $j_{s}$ are the individual slice specific convolution kernels derived from the single slice k‐spaces that may or may not contain slice‐specific FOV shifts. However, the main drawback of this slice specific reconstruction process is that it is limited to the separation of slices. In‐plane undersampling requires a separate reconstruction step. The analogue reconstruction procedure for two slices and R = 4 is sketched in Figure 6c.

An interesting property of the slice‐GRAPPA approach lies in the ability to quantify the so‐called slice‐leakage artifact “L”‐factor. This was introduced by Moeller et al 66 as an attempt to quantify and analyze interslice leakage artifacts as a quality metric in addition to the g‐factor noise enhancement. The original L‐factor estimation procedure in Moeller et al 66 uses Monte‐Carlo time series analysis in conjunction with frequency modulated small‐signal perturbations. Alternatively, as shown by Cauley et al 68 the L‐factor can be derived from the slice specific weights $K_{z}$ themselves which ideally pass only the slice z and cancel the contribution of all other slices. Thus, undesired residual signal contributions from other slices directly characterize the slice leakage. In addition, this property can be used to constrain the weights calibration such that contributions of all other slices are blocked when reconstructing a slice, known as split slice‐GRAPPA (SP‐SG). With the explicit constraint that the application of weights to reconstruct a specific slice cancels the contribution of all the other slices ( $j_{s}K_{z}=0$ for s≠z) the appropriate split‐slice GRAPPA reconstruction weights are:

(11) $$K_{z}=i_{z}\cdot j_{s}^{H}\cdot {\left(\overset{S}{\underset{s=1}{\sum }}j_{s}\cdot j_{s}^{H}\right)}^{-1}$$

It is important to note that the reduction of interslice leakage potentially comes at the cost of increased intraslice artifacts. These artifacts can be traded against each other by adding additional weighting parameters as described more fully in Cauley et al 68. It is in general important to note that slice‐leakage essentially is residual aliasing along the slice direction.

### Direct GRAPPA in 3D K‐Space

A further option for SMS‐CAIPIRINHA reconstruction with GRAPPA becomes evident when recognizing the 3D nature of the SMS acquisition 54, 55 and presenting the CAIPIRINHA phase cycles along the slice direction as a 2D reconstruction problem in analogy to volumetric 2D CAIPIRINHA 57 (Fig. 6b). This can be best imagined by understanding the multiband excitation as a volume excitation and the CAIPIRINHA RF‐phase cycles or gradient blips as k‐space phase encoding. The corresponding sampling positions in ky‐kz space for various blipped CAIPI schemes are depicted in Figure 4. Thus, as demonstrated for SMS EPI by Zhu et al 55, a 3D reconstruction kernel can be specified with source points according to the 2D CAIPIRINHA pattern and target points according to the missing k‐space locations within the 3D kernel region. Similar to standard GRAPPA, the required kernel can be calibrated within a low resolution 3D k‐space and finally applied to the acquired data to reconstruct all the missing points. The direct GRAPPA approach has great flexibility and similar to the SENSE‐GRAPPA hybrid performs the reconstruction in one single step. As only one kernel is used, trading off in‐plane and slice aliasing (slice‐leakage) is not possible. The direct GRAPPA approach in 3D k‐space can be set up to disentangle the slices from a single SMS acquisition, or alternatively from a complete volume, in one single step after appropriate reformatting of the raw data. In the latter case, full slice resolution for the calibration data is not required.

The question as to which of the presented methods/implementations is best suited to which imaging situation is difficult to answer and in many cases will dependent on the application or the preference of the observer. To our knowledge, at the time of this writing, a thorough quantitative comparison of the reconstruction performance is lacking, although to be expected at some future stage.

### Beyond SMS Aequisition with Cartesian Caipirinha

The CAIPIRINHA sampling considered so far aims for a controlled but coherent signal aliasing that maximizes the coil's ability to separate overlapping voxels. The use of FOV shifts causes characteristic aliasing patterns, hence, potentially sharp discontinuities in the reconstruction related noise enhancement. Some authors have recently advocated forms of less coherent aliasing, by pseudorandom RF phase cycle patterns in abdominal GRE acquisitions 69, or pseudorandomly structured blip patterns in EPI 70. The slice aliases are then more evenly “smeared out” over the entire phase encode FOV, resulting in a larger average reconstruction noise (g‐factor) than for CAIPIRINHA but with a smoother distribution.

Non‐Cartesian SMS acquisition schemes have also been proposed. One such method is radial CAIPIRINHA 71 where the RF phase cycling is performed across neighboring radial spokes. Other studies have investigated spiral readouts with application of z‐encoding blips during the spiral readout 72. The attractiveness of non‐Cartesian sampling schemes without a clearly defined phase‐encoding direction is that the coil sensitivity information along all three spatial dimensions can be exploited, allowing for improved image reconstruction, or higher undersampling factors than with the common Cartesian CAIPIRINHA sampling. Another approach in this direction was taken by Breuer et al 73 for single‐slice and 3D imaging, by applying oscillating “Zig‐Zag” phase‐encode gradients during the readout and signal acquisition to allow for additional exploitation of coil sensitivities along the read‐out direction in a similar manner to Moriguchi and Duerk 74.

Setsompop and colleagues recently extended this concept to SMS imaging under the name wave‐CAIPI 58, 75. Both Zig‐Zag CAIPIRINHA and Wave‐CAIPI sample at positions (ky,kz) of regular CAIPIRINHA but impart a further phase modulation during the readout by applying additional oscillating gradients simultaneously with the trapezoidal readout gradient, causing each voxel's alias to spread across the FOV along all three dimensions. In the case of Wave‐CAIPI, oscillating y and z gradients are played out with ¼ cycle between them, so as to create a corkscrew trajectory that together with the underlying 2D CAIPIRINHA pattern results in a high degree of voxel spreading. This results in significant g‐factor advantages over conventional CAIPIRINHA as recently demonstrated in wave‐CAIPI RARE acquisitions 58. Furthermore, unlike spiral trajectories that require computationally intensive regridding reconstructions, wave‐CAIPI has the favorable, and for non‐Cartesian schemes unusual, property that it can be reconstructed directly by treating the voxel point spread as a convolution in image space. A schematic of the technique is shown later in the section on SMS applications.

## PRACTICAL IMPLEMENTATION CONSIDERATIONS

This section discusses several practical aspects of the implementation and use of SMS acquisitions.

### Signal‐to‐Noise in SMS Acquisition

SMS acquisitions share the SNR benefits of 3D sampling that arise due to Fourier averaging. This improves SNR efficiency by $\sqrt{N}\;$compared with single slice acquisition as N times more spins are simultaneously excited. Consequently, *N* slices can be simultaneously acquired (in the same time as conventionally sampling a single slice) without any SNR penalty other than g‐factor reconstruction noise. As the use of SMS typically involves reductions in the repetition time well below the T1 of the tissue of interest, the available longitudinal steady state magnetization for signal formation will, however, be somewhat lower. SNR optimal acquisitions of short‐TR SMS EPI, therefore, require excitations at the Ernst angle, which has the beneficial side effect that RF peak power and SAR can be considerably reduced. Also in this regard, SMS has some of the favorable properties of 3D sampling.

### Gradient or RF Phase Encoding?

Gradient and RF phase encoding for effecting slice shifts in controlled aliasing are mathematically equivalent as discussed in the previous section, however, their applicability depends on the type of sequence. RF phase encoding is not feasible in echo‐train sequences that acquire multiple or all k‐space lines per excitation, which will, therefore, all carry the same phase. In single‐shot EPI or RARE/TSE sequences, any phase pattern between lines can, however, be achieved by the application of gradient blips between successive lines along the readout. Use of RF encoding in a single‐shot RARE type sequence is in principle possible by using the appropriate slice specific phase cycles by means of the refocusing pulses, but this is somewhat impractical, and more importantly, it will be incompatible with the Carr‐Purcell‐Meiboom‐Gill condition. For segmented echo train sequences, there is some potential for RF encoding but with limited flexibility, by cycling the RF phase between EPI segments 76 or shots in a RARE/TSE sequence as suggested in Norris et al 33. RF encoding also becomes nontrivial in steady‐state free precession (SSFP) sequences (irrespective of an echo‐train readout), because the RF phase cycling for slice encoding may not interfere with the RF spoiling scheme 65.

A further practical consideration is that the phase imposed by an RF pulse has no spatial dependence, whereas a z‐gradient blip always imposes a phase that is linearly proportional to the blip moment and distance away from the isocenter; therefore, there will be a constant shift between equidistant neighboring slices, while RF encoding in principle allows for any arbitrary individual phase schedule to be given to the simultaneous slices. For constant interslice shifts, it is always possible to replace RF encoding by gradient blips, and in many cases this may be easier to implement in the sequence code. It is self‐evident that more complex 3D aliasing patterns, e.g., due to spiral or wave‐CAIPI readouts can only be achieved by the use of gradients.

A further consideration is that the gradient blips will cause some through slice dephasing. This may generally be neglected for the typical slice thicknesses used in 2D imaging, but may be a consideration if SMS is applied to multislab 3D acquisitions.

### Calibration Data and Reconstruction

Just as standard pMRI, SMS acquisitions require reference data to successfully separate the simultaneous slice signals. In applications that acquire many volumes in a time series (e.g., fMRI, DTI), the reference data for slice reconstruction usually consists of a separate fully‐sampled multiple slice scan. In this situation, a high quality reference dataset can be obtained using the same acquisition sequence and readout module because it only takes up a small fraction of the total scan time. For structural (single volume) slice‐accelerated imaging, the acquisition of a fully sampled reference dataset with the same sequence is not an option. A practical way to acquire single‐slice reference data quickly, and without dead time and SNR loss due to magnetization preparation such as inversion recovery, is the use of FLASH calibration scans, as the reconstruction algorithms described in Section 3 are either independent of or very robust against differences in contrast between the imaging and calibration data 19, 68.

EPI acquisitions are prone to N/2 Nyquist ghosting artifacts, due to eddy current delays in the alternating readout gradients leading to nonalignment of the odd and even k‐space lines. Similar to the problematic interaction of residual N/2 ghosting with in‐plane acceleration factors that are multiples of two, this causes challenges for SMS reconstruction, where the Nyquist ghosting resembles, and hence becomes inseparable from, the intended effect of CAIPI slice shifts by FOV/2. The particular challenge for SMS is that ghosting cannot be fully corrected for, before unaliasing the slices, because the degree of N/2 ghosting varies by slice due to the spatial dependence of the eddy currents. As a solution, it was proposed in Setsompop et al 31 to acquire the SMS calibration data with the same level of ghosting as the SMS‐accelerated scan (i.e., by using the same sequence module as usually the case in EPI implementations for fMRI and DTI), and to then enforce robustness against ghosting by training kernels for the odd and even phase encode lines separately. Application of these kernels will unfold the slices correctly, and they can subsequently be ghost‐corrected using standard techniques as in conventional single‐slice EPI reconstructions. This separate odd‐even strategy is now widely implemented in the context of slice‐GRAPPA 31, but it can equally be incorporated into any of the other GRAPPA‐based SMS reconstructions.

For EPI calibration scans in case of in‐plane acceleration, it is desirable to match the echo‐spacing or the imaging and calibration scan for them to have the same phase evolutions and point spread (distortion). If this is not the case, the GRAPPA kernel or coil sensitivity profiles derived from the calibration data may not result in a clean reconstruction, causing residual artifacts particularly in regions of large B0 inhomogeneity. In a typical implementation, this is achieved by acquiring the ACS data in a segmented manner (with number of segments equal to the acceleration factor), at a TR time interval so as to remain in the steady state. A major concern here is motion or breathing occurring during such segmented reference acquisition, which can have a serious effect on the quality of the subsequent reconstruction. This has been addressed by using the FLEET technique (fast low‐angle excitation echo‐planar technique) 77, 78, which acquires all segments belonging to a given slice in immediate succession rather than seconds apart. Alternatively, FLASH type reference scans have been advocated to considerably improve the temporal stability in image time series, but often at the cost of slightly elevated residual aliasing compared with conventional segmented ACS. In a comparison of the different approaches at 3 and 7T, the FLEET method consistently exhibited lowest residual aliasing and highest temporal stability, across field strengths, acceleration factors, and spatial resolution 78. In case of in‐plane and slice acceleration, the strategies for acquiring reference data for slice and in‐plane reconstruction can be combined in several logical ways.

### Residual Aliasing and Slice Leakage

Similar to in‐plane pMRI, imperfect SMS reconstruction results in residual aliasing artifacts. In the context of SMS imaging, residual aliasing often originates from different slices and thus is sometimes referred to as “slice leakage.” This has received much attention especially in the context of EPI, because residual aliasing in SMS fMRI data is more apparent than in in‐plane accelerated scans. Any temporal disparity within a calibration dataset for slice and in‐plane unfolding can significantly reduce the quality of parallel imaging reconstructions as shown in the context of in‐plane acceleration by Polimeni et al 78. The manifestation of slice leakage is slightly different from the classical in‐plane case. The in‐plane artifact will typically look continuous, i.e., a consistent, ghost‐like shifted image overlaid on the correct image. In SMS imaging, the kernels for successive slices may be completely independent (with the exception of the 3D‐method described earlier). While one slice may show significant residual aliasing due to a corrupted kernel, another slice may be completely free from artifacts. This independent behavior makes the artifacts much harder to identify.

Finally, the impact of residual aliasing depends on the application. In anatomical imaging, residual in‐plane aliasing that originates from the same imaging plane is typically easy to interpret, and is unlikely to cause misinterpretations. SMS imaging, however, is currently most commonly used for (resting state) fMRI and diffusion imaging of the brain. These methods analyze data for connections across the entire brain and spurious correlations due to residual aliasing can easily be mistaken for long‐range connectivity.

### Image Reconstruction Times

The computational demand of SMS reconstructions is still an obstacle to some applications such as real‐time fMRI, and high‐resolution EPI acquisitions with high in‐plane and/or slice acceleration factors often suffer considerable reconstruction delays on standard vendor reconstruction hardware. This is being addressed by code optimization, GPU implementations, and use of high‐end hardware. In particular, the use of coil compression now plays an important role in effectively reducing the number of channels, thereby drastically lowering computational demand. This can be achieved by PCA approaches, or by explicitly exploiting the spatially varying coil sensitivities along the nonundersampled (the readout) encoding direction in Cartesian acquisitions 79. In this technique, known as geometric coil compression, the channel reduction is performed in image space at each spatial coordinate along the fully sampled dimension. Reduction ratios of 2–4 and higher have been demonstrated, with relatively minor penalty in SNR 79.

However, it remains important to take the computational burden into account when implementing SMS reconstruction. If implementation on the scanner for online reconstruction cannot easily be achieved, researchers might consider interfacing with external computational resources such as the Gadgetron framework 80, or off‐line reconstruction.

### Public Availability of SMS Sequences

To the authors' knowledge, no product implementations of SMS are being offered by the MRI vendors at the time of writing. However, the early phase of the Human Connectome project (http://www.humanconnectome.org) gave a boost to the acquisition techniques for fMRI and DTI, and has facilitated the development and dissemination of SMS acquisition within the neuroimaging community. While the availability of SMS implementations is still limited and the process of sequence‐ and reconstruction implementation often needs to be done in‐house, mature SMS EPI customer solutions for the dominant commercial platforms can be obtained from some research labs under sharing agreements, or as a third party commercial software add‐on.

## APPLICATIONS OF SMS ACQUISITION

**Figure 7:**
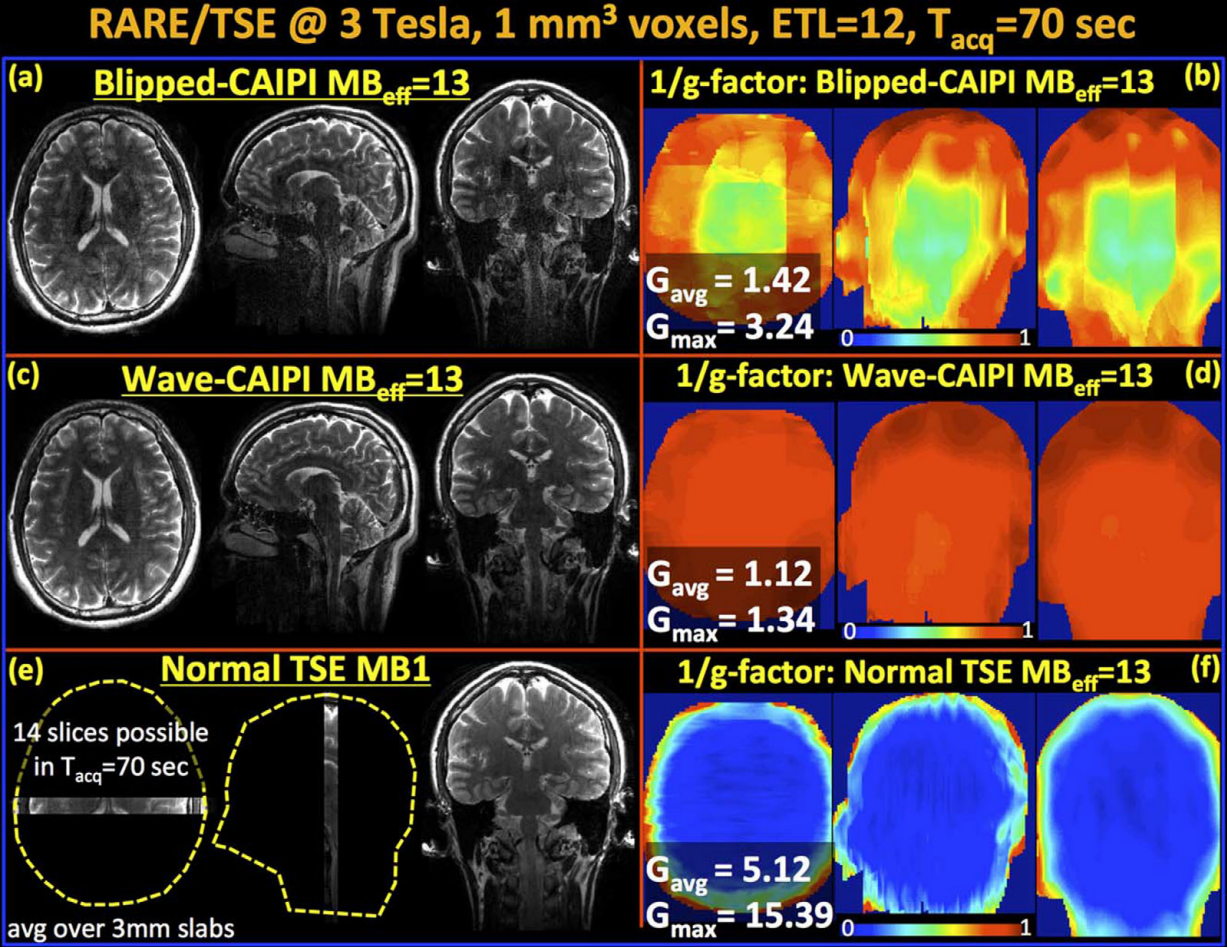
Reconstructed volumes and g‐factor analysis for MB‐15 RARE/TSE at 3T. Note, that because two slices remain outside the head, this leads to an effective MB factor of MB_eff_ = 13. **a,b:** Blipped‐CAIPI suffers from noise amplification especially in the middle of the volume with g_max_ = 3.24 and g_avg_ =1.42. **c,d:** Wave‐CAIPI yields high quality data and close to perfect SNR retention with g_max_=1.34 and g_avg_ = 1.12. **e:** Fully sampled MB‐1 product RARE/TSE acquisition is able to cover a very limited FOV (14 slices) in the same acquisition window. **f:** The 1/g‐factor analysis for MB_eff_ = 13 reconstruction without FOV shifting and Wave. All images (a,c,e) are scaled identically. [Reproduced with kind permission from Gagoski et al 58].

The obvious strength of SMS imaging is the acceleration of time critical applications, such as abdominal 81 or cardiac imaging 82, applications that acquire a time series for functional evaluation (fMRI, CE‐MRI), and acquisitions with very long acquisition time (DTI). Despite the benefits for these applications, the spread of SMS acquisitions into real clinical and research applications is still rather thin; first, because it is still a relatively young technique (despite some early publications as explained in the Introduction section), and, second, the implementation on the different vendor platforms is far from complete, as discussed in the previous section. The majority of reports using SMS techniques are currently focused on fMRI and diffusion, with a few recent reports that apply SMS in perfusion imaging, both ASL based 83, 84 as well as dynamic susceptibility based 85, and angiography 86. The main organ investigated remains the brain, with the spread to other organs also being somewhat delayed. The significant speedups possible with SMS have also triggered the application of SMS to TSE/RARE type acquisitions for neuroimaging to reduce the lengthy acquisition times involved in T2‐weighted anatomical imaging, and also to demonstrate the potential reduction in RF power deposition. By using PINS pulses for excitation and refocusing, Norris et al 34 could acquire TSE of the whole brain with 1 mm in‐plane resolution and 2‐mm‐thick slices in 2 min without being restricted by SAR. A recent report by Gagoski et al 58 achieved thinner slices by the use of Multi‐PINS giving a 1 mm isotropic resolution, and increased the attainable acceleration (effective MB factor of 13) even further by the addition of the WAVE‐CAIPI technique (Fig. 7).

In the following, we give an overview of the major research efforts to date that have used SMS acquisition, namely in the domains of abdominal and cardiac imaging, fMRI and DTI. For the interested reader, we note that several studies have been presented at the major conferences, but at the time of writing they were not available as full journal publications.

### Abdominal and Cardiac Imaging

**Figure 8:**
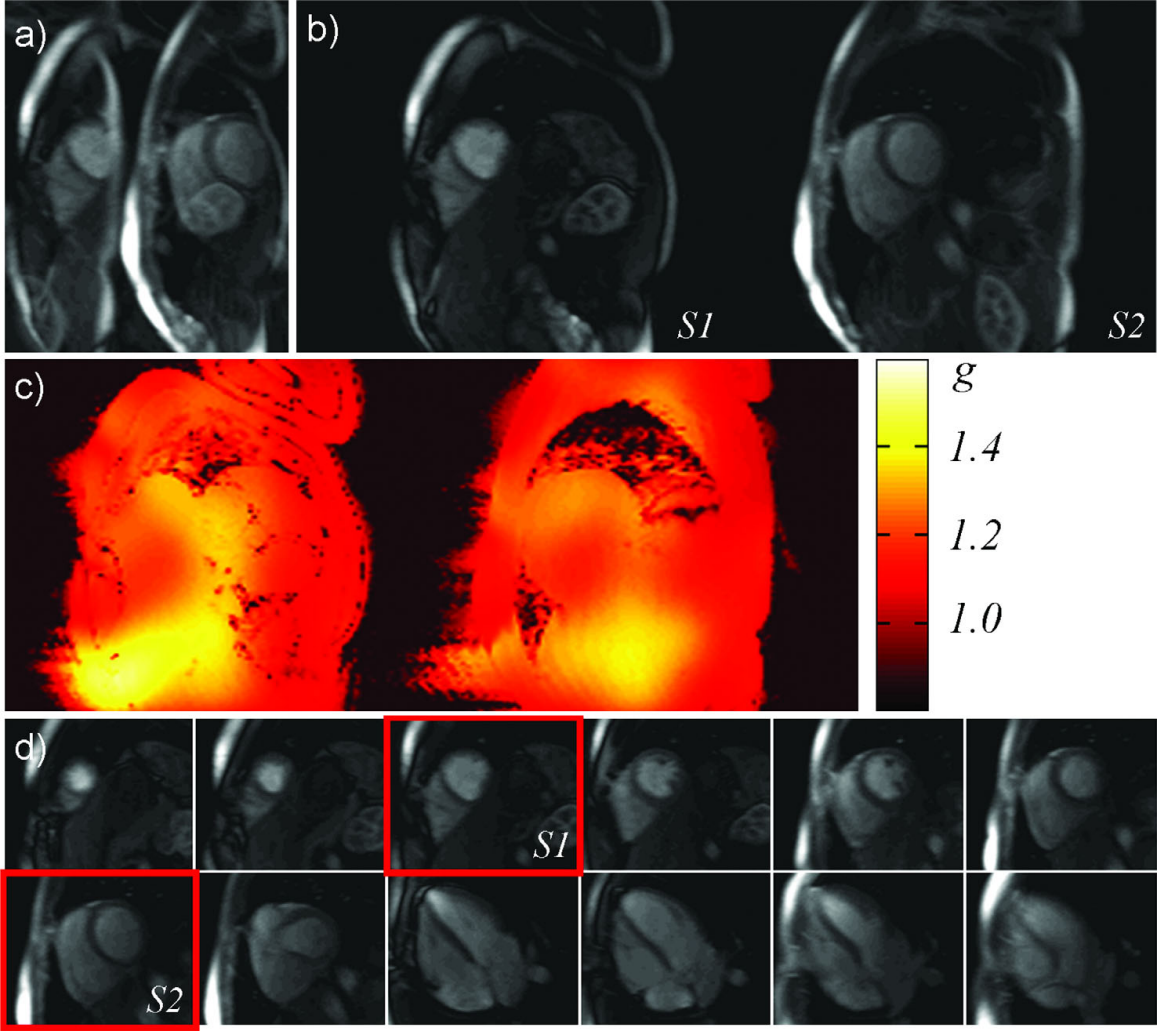
In vivo 12‐slice myocardial perfusion imaging. **a:** Simple Fourier transform of one measurement, showing the simultaneous acquisition of two short axis slices. **b:** Reconstruction of the simultaneously excited slices shown in (a). **c:** G‐factor maps for the reconstruction shown in (b). **d:** Enlarged sections of all 12 slices acquired during one first pass experiment. Two slices each were acquired at the same time. The passage of the contrast agent through the myocardium is shown in eight short and four long axis slices. The red boxes indicate the two simultaneously excited slices shown in (a) and (b). (Reproduced with kind permission from Stäb et al 65.

Cardiac and abdominal imaging are quite time critical applications. For cardiac MRI, this is due to the necessity to acquire data during a single breathhold or even during a single heartbeat, so SMS imaging lends itself naturally due to the large SNR advantage and increased coverage compared with standard pMRI in cardiac perfusion MRI. Also other sequence types and applications for cardiac MR profit, such as saturation‐recovery prepared cardiac perfusion which is not affected by slice overlap, and cardiac stress examinations, where the heartbeat is faster and breathhold periods can become very short. Unfortunately, SSFP based sequences are limited by SAR restrictions due to the fast TR and only relatively low MB factors can be applied. However, CAIPIRINHA‐based techniques (see Figure 8) have been shown to significantly increase imaging speed and volume coverage for myocardial perfusion MRI and real‐time and cine SSFP MRI 65, 87. It is important to note that, as the majority of sequences for cardiac MRI are non‐EPI based, the acquisition of reference data follows different strategies and can be acquired over more than one heartbeat [TGRAPPA 88] or even without triggering. A recent publication showed the potential to perform cardiac DTI at 3T using SMS imaging 82. Multislice (MS)‐CAIPIRINHA was actually first shown in abdominal imaging 13, but there are currently not many clinical studies as receive coils with many elements for body MRI have only recently become available, and also because of the slow implementation of the technique. A recent study, however, has demonstrated a slightly modified SMS acquisition by acquiring image and navigator slices simultaneously 89.

### fMRI

There is a clear rationale for accelerating fMRI acquisitions as more time points increase statistical power. This can be done by either using blipped‐CAIPI sampling to allow MB factors as high as 16 or by combining SMS with simultaneous image refocusing (SIR) 17, 26, 27, 90. The latter, however, requires an increase in the EPI echo spacing, which results in a lower sampling bandwidth and increased geometric distortions in the phase encoding direction. Alternatively, SMS‐fMRI also increases the flexibility with regard to increasing spatial resolution while still permitting short volume acquisition times 15, 17. The use of in‐plane parallel imaging made multiecho EPI (ME‐EPI) a viable technique for fMRI 91 as multiple images could be acquired over a relevant range of TR values. The extension to multiband multiecho, hence, still also requires in plane acceleration and has been shown to have improved sensitivity over multiecho acquisition for resting state fMRI at 3T 92, and for both resting state and task fMRI at 7T 93.

In addition, the increase in the (temporal) degrees‐of‐freedom allows the application of advanced analysis strategies such as temporal independent component analysis or ICA 94, 95, as well as a denser temporal sampling of physiological fluctuations in the fMRI signal 96, which helps in their identification and reduction during the analysis 97. Even in the extreme case of a single thick slab as used in inverse imaging, blipped‐CAIPIRINHA can be used to improve these ultrafast acquisitions schemes 98. One should be aware that in practice an adaptation of the analysis strategy needs to be considered to get the most out of the increased sampling rate. This could be the measurement and incorporation of physiological signals (heart beat and breathing), or by using a statistical analysis which uses a corrected degree of freedom 99 as the autocorrelation structure of the noise is modified in the faster sampled time course 17.

A detailed assessment of the benefits for fMRI of using SMS is not straightforward as many parameters influence performance. One would have to include the benefits of the excitation of a larger volume (as mentioned in the Introduction section) and the larger number of time points, which are counteracted by the noise introduced during reconstruction as already described in the section Reconstruction Methods. Furthermore, a reduced magnetization is available due to the much shorter TR compared with tissue T1 even if the optimal flip angle (Ernst angle) is used, which impacts the image SNR. To what extend this reduces the temporal SNR in fMRI acquisitions depends on the relative contribution of physiological noise: typical fMRI acquisitions are physiological noise dominated, allowing rather high MB acceleration factors 90 while maintaining high tSNR. It has also been pointed out that, in the case of very high MB factors, the temporal noise correlations between un‐aliased voxels can cause bias in fMRI resting state 100. SMS‐fMRI can also lead to a distinctive multislice pattern as found in a temporal ICA analysis 95, potentially due to residual aliasing between slices, however, recent developments in SMS reconstruction should be able to considerably mitigate this issue 68.

The SMS approach is particularly interesting for SE‐EPI in fMRI, as this is an inherently slow method that needs a much longer TE (approximately two‐fold) compared with GE for optimal SE BOLD contrast 101, 102, 103. The SMS acceleration can reduce volume repetition times to 2–3 s or less, even for high resolution acquisitions and whole brain coverage, thus allowing adequate sampling of the event‐related BOLD response. However, similar to DWI (see below), the refocusing pulses needed in addition to the excitation pulses increase SAR, which in turn results in prohibitively long volume TRs at ultrahigh field strengths, where SE‐EPI is most relevant due to the BOLD contrast characteristics 104, 105, 106, 107, 108. Two studies have shown that using PINS pulses can help to overcome the limitations, either using them as excitation and refocusing pulses 109 or in combination with standard MB pulses for excitation and PINS pulses for the power‐intensive refocusing pulses 110.

### Diffusion‐Weighted Imaging

Most modern diffusion weighted imaging is performed with DW‐EPI. It was, hence, one of the core beneficiaries alongside fMRI from the advent of SMS imaging 31. As with fMRI, the potential benefits are clear, namely a further decrease in the acquisition times. However, in several respects, the considerations in performing DW‐EPI are different than for fMRI. First, DWI is an insensitive technique, dominated by thermal SNR. Second, as a spin‐echo based technique, both excitation and refocusing pulses need to have multislice properties which can result in high peak voltages for the refocusing pulse. Especially at high field strength, high power deposition can also become a problem. Third, the DWI experiment is highly sensitive to motion, which in SMS imaging may affect each slice differently, leading to potential problems with the reconstruction for motion that is more complex than simple displacement. To date, slice acceleration factors used have been typically three for DWI as compared to eight for fMRI, as this ensures low g‐factor noise. For example, the HCP protocol at 3T uses an MB factor of 3, 6/8 partial Fourier, and no in‐plane acceleration for an isotropic resolution of 1.25 mm. At 7T, the in‐plane resolution is 1 mm, but it is then necessary to use an in‐plane acceleration of a factor of 3 to keep distortion to an acceptable level, while reducing the slice acceleration factor to 2 25, 43.

To reduce peak power, it has been proposed to split the refocusing pulse into two sequential but overlapping pulses 24, as described in the section on RF pulses. The main alternative to this approach is to use PINS or multi‐PINS pulses for the refocusing, combined with conventional multiband excitation pulses. This concept made it possible to obtain high spatial resolution DWI data at 7T using both a zoomed 39 and a standard whole brain approach 40. An alternative to zooming using orthogonal slice selective gradients is to use RF pulses that are spatially selective in two dimensions and periodic in the third dimension, thus automatically lending themselves to SMS imaging. These have been successfully implemented for imaging the spinal cord by combining SMS excitation with a Hadamard encoding scheme 111.

## CONCLUDING REMARKS

This review has attempted to provide a comprehensive snapshot of the state of the art of SMS methodology, with particular emphasis on SMS excitation, reconstruction, and applications. Much of the material presented will remain valid and relevant, but as SMS is anticipated to be a dynamic and rapidly developing field for some time, further important developments can be expected. With vendors rapidly adopting the technique, its establishment in routine clinical application in the near future seems certain. A series of first studies on SMS cardiac imaging has shown great promise for applications below the neck 87.

It was not until it was proven beneficial for fMRI in 2010 17 that SMS suddenly took off in the neuroimaging community in a manner similar to the early days of parallel imaging. Seldom has an MRI technique been developed to a high level of maturity so quickly, and received such rapid acceptance, as has the application to EPI based BOLD fMRI and DW‐MRI where it has effectively established itself as a new standard. Much of the initial push can be attributed to the Human Connectome Project, and now a much broader base of researchers continues to work on improving SMS techniques.

The recent literature has indicated that parallel‐transmit technology will play an increasingly important role, especially at ultrahigh field strengths. A further aspect of practical relevance will remain the refinement of suitable reference scans for calibrating the reconstruction, as the quality and integrity of the calibration data are essential especially for highly undersampled scans.

In conclusion, SMS imaging can offer both significantly accelerated data acquisition, and reduced RF‐power deposition for a broad range of MRI applications. Its position as a cornerstone of modern MRI seems assured.
